# Parents’ Perceptions Regarding Their Children’s Medications and Expert-Assessed Drug-Related Problems in Pediatric Patients with Inborn Errors of Metabolism

**DOI:** 10.3390/children10121873

**Published:** 2023-11-29

**Authors:** Tanjana Harings, Martina Patrizia Neininger, Simone Eisenhofer, Alena Gerlinde Thiele, Wieland Kiess, Astrid Bertsche, Skadi Beblo, Thilo Bertsche

**Affiliations:** 1 Institute of Pharmacy, Clinical Pharmacy, Medical Faculty, Leipzig University, Bruederstrasse 32, 04103 Leipzig, Germany; tanjana.harings@uni-leipzig.de (T.H.); simone.eisenhofer@uni-leipzig.de (S.E.); thilo.bertsche@uni-leipzig.de (T.B.); 2 Drug Safety Center, Leipzig University and University Hospital, Bruederstrasse 32, 04103 Leipzig, Germany; 3 Center for Pediatric Research, University Hospital for Children and Adolescents, Liebigstrasse 20a, 04103 Leipzig, Germany; alena.thiele@medizin.uni-leipzig.de (A.G.T.); wieland.kiess@medizin.uni-leipzig.de (W.K.); astrid.bertsche@uni-greifswald.de (A.B.); skadi.beblo@medizin.uni-leipzig.de (S.B.); 4 Division of Neuropediatrics, University Hospital for Children and Adolescents, Ferdinand-Sauerbruch-Strasse 1, 17475 Greifswald, Germany

**Keywords:** adverse drug reaction, drug-related problems, inborn errors of metabolism, inherited metabolic disease, medication, parents, pediatric, perceptions

## Abstract

We aimed to explore parents’ perceptions of their children’s medication use for inborn errors of metabolism (IEM), including the importance of medication intake, potential complications, and concerns about adverse drug reactions (ADR). Additionally, we aimed to determine expert-assessed clinically relevant drug-related problems, particularly those attributable to IEM. We interviewed 108 parents of 119 pediatric patients with IEM using a questionnaire relating to their perceptions regarding their children’s IEM medication. In affected siblings, a questionnaire was used for each child. We performed medication analyses to evaluate the patient’s complete medication regimen for clinically relevant drug-related problems, including medication for conditions other than IEM. It was very important to the parents of 85% of the patients to use IEM medication exactly as prescribed. The parents of 41% of patients perceived complications in their children’s use of IEM medication. The parents of 47% of patients reported fears concerning ADR because of IEM medication. Parents observed ADR in 27% of patients because of IEM medication. In 44% of patients, medication for conditions other than IEM was inadequate because of drug-related problems not associated with the IEM; a safe alternative existed in 21% of patients. In summary, almost half of the parents of patients with IEM reported complications with their child’s IEM medication intake and fears of ADR. Medication analyses showed that drug-related problems occurred regardless of IEM, emphasizing the general need to prescribe and dispense adequate, child-appropriate medication to minimize clinically relevant drug-related problems in pediatric patients.

## 1. Introduction

Inborn errors of metabolism (IEM) include a broad spectrum of rare diseases. Up to 1894 diseases are currently defined as IEM, and about one in 500 newborns is affected by one of the known IEM [1,2,3,4,5,6,7]. In Germany, IEM patients are often detected in the first days of the child’s life in a newborn screening, which currently tests for 17 target diseases, 12 of which are metabolic diseases [1,6,7]. In the case of abnormal findings in the newborn screening, an extended specific screening for the suspected disease is carried out to confirm or refute the preliminary diagnosis [7]. Early diagnosis, facilitated by newborn screening, and prompt initiation of therapy are crucial for a good prognosis and survival of many patients [1,8]. Further, the life expectancy of patients with IEM increased during the last decades owing to improved diagnostics and treatment [9,10]. Treatment of the IEM often requires normalizing metabolites through substrate reduction and product supplementation [11,12,13,14]. Consequently, patients often must follow a special diet and take medicinal products or nutritional supplements regularly. Limited research on IEM exists, with a focus on phenylketonuria, as it is considered a model disease. It was the first IEM to be screened (since 1962), so the body of experience is the greatest. Previous studies have focused, for example, on the impact of the diseases on quality of life, affected executive functions, or medical treatment needs [15,16,17,18,19,20,21]. However, there is a lack of data on parental perceptions regarding their children’s IEM medication across the broad spectrum of IEM. Yet, those perceptions, such as complications with medication intake or fears of adverse drug reactions (ADR), can influence the parents’ attitudes toward the child’s IEM medication. They should be taken into consideration when counseling affected patients and their parents because, depending on the condition, incorrect medication intake could significantly worsen the child’s health [1,10,22,23]. We also hypothesized that pediatric patients with IEM are particularly susceptible to drug-related problems with medication for conditions other than IEM because of potentially problematic excipients.

Thus, we investigated parental perceptions about their children’s IEM medication. These insights into parents’ perceptions could contribute to improving patient safety. We also aimed to identify clinically relevant drug-related problems due to IEM with medication for other conditions. This analysis can provide valuable information on medication selection and counseling, especially for physicians and pharmacists who are not specialized in IEM but treat children with IEM and dispense medication for them.

## 2. Materials and Methods

### 2.1. Study Design, Participants, and Setting

In this exploratory status quo survey, we interviewed parents of pediatric patients by telephone about their perceptions regarding their children’s IEM medication using a questionnaire. Additionally, the complete medication of each patient was analyzed for potential drug-related problems. We analyzed both the medication for the IEM (IEM medication) and the medication for other diseases available at the patient’s home. The term “IEM medication” includes both medicinal products as well as other substances used to treat IEM that are referred to as “nutritional supplements”, such as vitamins (ATC Group A11), mineral supplements (ATC Group A12), amino acids, and other miscellaneous substances without Anatomical Therapeutic Chemical Classification (ATC-Code), such as corn starch (Supplementary Table S1). The term “medication for other chronic illnesses” refers to medication that is regularly taken or used for chronic illnesses other than IEM, e.g., medication for asthma (Supplementary Table S2). “As-needed medication” refers to medication that is taken for a short period of time for temporarily acquired illnesses, such as cold medication (Supplementary Table S3). The interview was conducted by telephone to ensure that the parents had their child’s complete medication available. We consecutively invited all parents of patients diagnosed with IEM who had an appointment at the pediatric outpatient clinic for metabolism disorders of a university hospital to participate in the study from May 2020 to September 2020. The inclusion criteria were the presence of an IEM in the patients and the sufficient ability of the parents to understand the questions and conduct an interview. The study was approved by the Ethics Committee at the Medical Faculty, Leipzig University, Germany (date: 17 December 2019, No 512/19-ek). Written informed consent was obtained from all participating parents who had to be the legal guardians of the pediatric patients when they had an appointment at the pediatric outpatient clinic. Parents with more than one affected child were asked to participate in the survey for each eligible child. Parents with more than one enrolled child were evaluated only once for their sociodemographic data.

### 2.2. Telephone Interview

We performed a structured telephone interview consisting of predefined questions (Supplementary Table S4). The following issues were addressed:

1. Parental perceptions regarding their children’s IEM medication; only for parents of children taking IEM medication;
2. The patient’s complete medication, prescribed by physicians or bought by themselves: IEM medication, medication for other chronic conditions, if applicable, and other as-needed medication (e.g., for colds);
3. Sociodemographic data of parents and patients.

The intensity of different constraints was assessed using a Likert scale ranging from 0 to 5 (0: I absolutely disagree; 5: I completely agree). We pretested the interview for comprehensibility with 10 pharmacists and adjusted the questions accordingly; in particular, the presentation of the Likert scale was improved. Answers to open questions were clustered by the interdisciplinary study team consisting of pediatricians -among them physicians specialized in IEM-, pharmacists, and nutritionists to make the answers accessible for quantitative evaluation.

### 2.3. Expert-Assessed Drug-Related Problems

A drug-related problem is defined as “an event or circumstance involving drug therapy that actually or potentially interferes with desired health outcomes” [24]. We evaluated the patients’ complete medication reported in the interview for potential drug-related problems, regardless of whether the medications were prescribed by physicians or purchased by the parents themselves or whether they were used for IEM or other acute or chronic conditions. The parents were asked to name their children’s (i) IEM medication (Supplementary Table S1), (ii) medication for chronic illnesses other than IEM (Supplementary Table S2), and (iii) as-needed medication (Supplementary Table S3). Pharmacists analyzed for each reported medication whether the indication as reported by the parents, dosage form, and excipients were appropriate for the respective age and IEM of the patients. Furthermore, the medications were reviewed for contraindications, interactions, or other implications due to the child’s condition. The medications with identified drug-related problems were evaluated by the interdisciplinary study team. As members of the study team were also involved in the treatment of the affected children, comprehensive information on the clinical condition and medical history of the patients was also considered in the assessment of the medications.

The medications were classified into five groups based on the nature of the identified drug-related problems:

1. Medication appropriate: The medication can be safely used in the patient, according to the literature;
2. Medication problematic without clinical relevance: According to the literature, the medication should not be used in the patient. However, the identified problems were negligible in the affected patients in our study. Yet, it cannot be ruled out that problems might occur in other patients;
3. Necessary medication with clinically relevant problems because no safe alternatives are available: According to the literature, the medication should not be used in the patient due to potential clinically relevant problems. However, the medication is necessary for the patient, and there is no safe alternative;
4. Non-necessary medication with clinically relevant problems because safe alternatives are available: According to the literature, the medication should not be used in the patient due to potential clinically relevant problems. Safe alternatives exist, or the medication is not necessary for the patient and can be omitted. Thus, those medications are avoidable;
5. Not assessable due to lack of specifications: The manufacturer or the parents did not provide sufficient information on the medication, so it could not be included in the analysis.

### 2.4. Analysis and Statistics

Frequencies are reported as numbers and percentages; continuous data are presented as median with first (25%) and third (75%) quartile (Q25/Q75) and range (min/max). Results were analyzed separately by age (patients aged < 6 years [Patients < 6 years] and patients aged ≥ 6 years [Patients ≥ 6 years]) to account for children’s increasing age. The average age at school enrollment was chosen as the age cut-off to determine potential differences as children become more independent. For the evaluation of feared and experienced ADR of IEM medication, we distinguished nutritional supplements, including vitamins, amino acids, mineral supplements, and various substances without ATC-Code from medicinal products. We hypothesized that parents might perceive those nutritional supplements to have a lower potential for ADR.

## 3. Results

### 3.1. Characteristics of Patients and Their Parents

During the study period, 152 parents of children with IEM were invited to participate. Of those parents, 118 (76%) agreed to participate and gave their written informed consent. Yet, 10 parents had to be excluded either because they withdrew their agreement later (4/152, 3%) or could not be contacted at the appointed interview date even after sending a reminder (6/152, 4%). Thus, 108 parents of 119 children were enrolled (Table 1), 99 (92%) with one child with IEM, 7 (6%) with two children with IEM, and 2 (2%) with three children with IEM.

Interview Section 1 was conducted only with parents of patients taking IEM medication; this applied to 96/119 (81%) patients [Patients < 6 years: 40/47 (85%); Patients ≥ 6 years: 56/72 (78%)]. The parents of five patients could not name their children’s medications at the time of the interview, so a medication analysis was not possible. Therefore, interview Section 2 was conducted with the parents of 114/119 (96%) patients (Patients < 6 years: 45, Patients > 6 years: 69).

### 3.2. Parental Perceptions Regarding Their Children’s IEM Medication

#### 3.2.1. Importance of IEM Medication Intake

**Figure 1:**
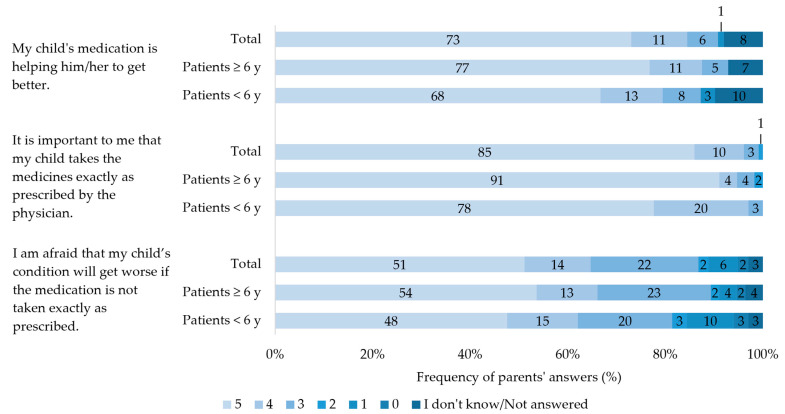
Parent’s level of agreement with given statements on their children’s medication. Patients < 6 years: Patients aged < 6 years. Patients ≥ 6 years: Patients aged ≥ 6 years. Scale ranging from 0 = I strongly disagree to 5 = I strongly agree. Percentages may not total 100 due to rounding.

In total, 73% of parents strongly agreed with the statement, “My child’s medication is helping her/him to get better”, and 85% strongly agreed with the statement, “It is important to me that my child takes the medicines exactly as prescribed by the physician” (Figure 1). Of the parents, 51% strongly agreed with the statement, “I am afraid that my child’s condition will get worse if the medication is not taken exactly as prescribed”.

#### 3.2.2. Perceived Complications with IEM Medication

According to the parents, 13/40 (33%) of the younger patients, Patients < 6 years, and 26/56 (46%) of the older patients, Patients ≥ 6 years, had perceived complications taking their IEM medication (total: parents of 39/96 patients, 41%). The most frequently stated complication in Patients < 6 years was the refusal to take the medication (9/40 patients, 23%; Table 2), while the most common complication in Patients ≥ 6 years was forgetting to take the medication sometimes (20/56 patients, 36%).

#### 3.2.3. ADR of IEM Medication as Perceived by the Parents

The parents of 22/40 (55%) Patients < 6 years and of 23/56 (41%) Patients ≥ 6 years stated that they feared ADR of their children’s IEM medication (total: parents of 45/96 patients, 47%). In detail, the parents of 9/40 (23%) Patients < 6 years feared ADR from medicinal products and 13/40 (33%) from nutritional supplements. In Patients ≥ 6 years, 12/56 (21%) feared ADR from medicinal products, and 11/56 (20%) from nutritional supplements.

According to the parents, 12/40 (30%) Patients < 6 years and 14/56 (25%) Patients ≥ 6 years had already experienced ADR from their IEM medication (total: 26/96 patients, 27%). In detail, 4/40 (10%) Patients < 6 years had already experienced ADR from medicinal products and 8/40 (20%) from nutritional supplements. In Patients ≥ 6 years, 4/56 (7%) had already experienced ADR from medicinal products, and 10/56 (18%) from nutritional supplements.

The feared and experienced ADR are shown in Table 3. The parents of one patient decided to discontinue a medication owing to fear of ADR and to avoid “further reduction of the child’s quality of life”.

### 3.3. Expert-Assessed Drug-Related Problems

The medication analysis was performed in 45 Patients < 6 years and 69 Patients ≥ 6 years, considering 884 medications (Supplementary Tables S2–S4). The medication of 28/45 (62%) Patients < 6 years and 36/69 (52%) Patients ≥ 6 years was assessed as appropriate (total: 64/114 patients, 56%), affecting 711/884 (80%) medications (Table 4). In Patients < 6 years, drug-related problems were identified in 17/45 (38%) patients, although safe alternatives would have been available in 12 patients (27%). In 33/69 (48%) Patients ≥ 6 years with drug-related problems, this applied to 12 patients (17%). A total of 90/884 (10%) medications could not be evaluated because the manufacturer did not provide sufficient information (70/884 medications, 8%) or the parents were not able to give sufficiently detailed information (20/884 medications, 2%).

## 4. Discussion

The interviews with parents of pediatric patients with IEM provided a comprehensive insight into the perceptions regarding IEM and their medication. A considerable number of parents perceived complications with their children’s IEM medication. The main problem reported in patients aged younger than 6 years, Patients < 6 years, was the refusal to take the IEM medication, whereas in patients aged 6 years onwards, Patients ≥ 6 years, the most common problem was forgetting to take the IEM medication, according to the parents. In addition, the number of parents who feared ADR from IEM medication was higher than the number who reported experienced ADR. The medication analysis showed that one in four Patients < 6 years and almost one in five Patients ≥ 6 years had inadequate medication at home for which there was a safe alternative. This was especially true for medication used for conditions other than IEM. Thus, the drug-related problems found in the medication analyses occurred because the patients were children, not because of the IEM. To alleviate drug-related problems in the pediatric population, more detailed counseling should be provided by physicians and pharmacists, regardless of whether an IEM is present in the patient.

### 4.1. Parental Perceptions Regarding Their Children’s IEM Medication

#### 4.1.1. Importance of IEM Medication Intake

Most parents were very concerned that their children take their IEM medication as prescribed by the treating physician. From previous studies, it is known that both parents and patients are very well educated about IEM and know that, depending on the condition, incorrect medication intake could significantly worsen the child’s health [1,10,23]. This was also reflected in our study, as about half of the parents were very afraid that their child’s condition would worsen if the medications were not taken exactly as prescribed.

#### 4.1.2. Perceived Complications with the IEM Medication

Nevertheless, about one-third of parents of Patients < 6 years and almost half of the parents of Patients ≥ 6 years reported problems with IEM medication intake, mainly due to patients’ refusal to take the medication and forgetting individual doses. Despite improvements in taste and a wider range of products, nutritional therapy is a major burden for many patients, which negatively affects their compliance with treatment [25,26,27]. Possibly, patients would be more accepting if there was more patient involvement and more child-centered education [28]. However, as the refusal of intake was mentioned mainly in Patients < 6 years, the possibilities of integrating the child into the therapy are limited [29]. Forgetting to take medication may indicate difficulties in integrating medication into daily life, which was also described as a problem by some parents. The problem was mentioned more frequently in Patients ≥ 6 years. Similar complications were also found in another study in which pediatric patients and their parents with various chronic illnesses (e.g., epilepsy, type 1 diabetes mellitus, acute and chronic respiratory diseases) were asked about difficulties with medication intake [30]. For patients aged younger than 6 years, the most common problem was the acceptance of medication intake, e.g., due to bad taste; for patients aged 6 years or older, the most common problem was adherence to the time interval, e.g., forgetting to take the medication [30]. Thus, the reported complications occur not only in IEM patients but in pediatric patients in general. With increasing age, pediatric patients become more independent and self-reliant regarding medication intake [31,32]. Thus, healthcare professionals should provide support and age-appropriate information about the importance of regular medication intake and the consequences if this is not achieved [20].

#### 4.1.3. ADR of IEM Medication as Perceived by the Parents

IEM therapy mainly involves nutritional supplements such as maltodextrin, levocarnitine, or amino acid mixtures rather than medicinal products. Due to the classification as a nutritional supplement, it could be assumed that ADR and their severity might be limited [33]. However, studies of high evidence and quality on the use of nutritional supplements are scarce, and in particular, there is a lack of literature on ADR [33]. Physicians’ therapy recommendations are often derived from a basic understanding of the metabolic processes involved and are described in the literature [13,14,33]. Rarely are therapy recommendations based on guidelines, as these exist only for very few IEM. Nevertheless, parents of about half of the children and adolescents reported fears of ADR, including very serious ones, such as organ damage or cancer. Known ADR of IEM medication are mainly derived from individual case reports, which were very rarely serious [33]. According to the parents, a quarter of the patients in our study had already experienced ADR, mainly from nutritional supplements, and mainly gastrointestinal ADR. This was surprising but plausible because gastrointestinal ADR are known to be the main ADR of nutritional supplements, such as maltodextrin or amino acid mixtures [33]. However, hardly any serious ADR occurred. Similar results were obtained in a study on parents of pediatric patients with epilepsy that also showed that feared ADR were more serious than experienced ones [22]. Nevertheless, these fears should be taken seriously by healthcare professionals. Better counseling on ADR of IEM medication by physicians and pharmacists may contribute to alleviating those fears of serious ADR and strengthen adherence.

### 4.2. Expert-Assessed Drug-Related Problems

Almost half of the patients had inappropriate medication at home. The problems identified refute our hypothesis that drug-related problems occur mainly because of IEM. The identified problems were mainly owing to the patient being a child, regardless of the underlying disease. This is also shown in another study [30]. Strikingly, about half of the medications that could not be included in the medication analysis due to a lack of information from the manufacturer were used for the treatment of IEM. This lack of information underlines the need for high-quality studies on the long-term use of nutritional supplements in IEM to determine potential risks for the affected patients.

Medications classified as “problematic medication without clinical relevance” included those in which patients received oral dosage forms that were too large for their age, according to the literature. For children under 12 years of age, only solid dosage forms smaller than 7 mm are considered safe to be taken [34,35,36,37]. However, the patients in this study had no problems swallowing solid dosage forms larger than 7 mm. Nevertheless, when prescribing or dispensing medication for children, care should be taken to ensure that appropriate dosage forms are chosen.

Medications classified as “non-necessary medication with clinically relevant problems, because safe alternatives are available” affected Patients < 6 years and Patients ≥ 6 years. The medications in question were not related to the treatment of IEM but to other conditions that occur in the general pediatric population. Also, the clinically relevant drug-related problems occurred independently of IEM. Safe alternatives existed for all medications in this category. When prescribing or dispensing medication to children, physicians and pharmacists should ensure that the medication is appropriate for the child’s age or comorbidities. The different physiology, pharmacology, and pathophysiology of children compared to adults demand special requirements for pediatric medication [38,39]. Particularly in chronically ill children, who may already be suffering from restrictions owing to their specific disease, care should be taken to ensure that only appropriate medication is used. However, some medications specifically approved for children contain ingredients that are inappropriate for children. This makes the selection of appropriate medication even more difficult. For this reason, problematic excipients and additives should already be avoided or reduced to a minimum during the development and authorization process for medicinal products that are to be used in children and adolescents.

### 4.3. Strengths and Limitations

Since the study was conducted in a special outpatient clinic for rare metabolic diseases of a university hospital, patients with a broad spectrum of IEM were included. This means that patients with very rare IEM with a prevalence of 1:1,000,000 are part of the study. The diversity of IEM in our study is very high compared to studies from the literature, which often focus on a single IEM.

Generalizability might be limited as the study was conducted in only one university hospital. Potential influences on the answers, such as social desirability, cannot be completely ruled out.

## 5. Conclusions

Our findings on complications with IEM medication intake show that forgetting and refusing to take medication are the main causes of difficulties in the treatment of IEM, although we also showed that regular intake of their children is very important to parents. Furthermore, the parents of half of the patients expressed fears regarding ADR caused by their children’s IEM medication, whereas ADR were experienced by one-quarter of the patients as reported by the parents. The expert-assessed drug-related problems identified in the medication analysis related in the fewest patients to their IEM, but mainly to being a child. A considerable number of the patients had medications in the household that were contraindicated due to patient age, ingredients, indication, or comorbidities. Consequently, pediatric specificities should be better considered when selecting medication, especially in chronically ill pediatric patients, to ensure that only safe medication is prescribed or dispensed.

## Figures and Tables

**Table 1:** Characteristics of patients and parents.

Characteristics	Patients Aged < 6 Years	Patients Aged ≥ 6 Years	Total
Number of parents [N]	44 *	66 *	108 *
Median age of parents (Q25/Q75; min/max) [years]	35 (32/37; 24/45)	41 (38/45.5; 30/54)	38 (34/44; 24/54)
Sex of respondent [N (%)]			
Male	5 (11)	11 (17)	16 (15)
Female	37 (84)	55 (83)	92 (85)
Number of children [N]	47	72	119
Median age of child (Q25/Q75; min/max) [years]	3 (1/5; 0/5)	12 (9/15; 6/17)	8 (3.5/13); 0/17)
Sex of child [N (%)]			
Male	29 (62)	43 (60)	72 (61)
Female	18 (38)	29 (40)	47 (39)
Diagnosis [N]			
Phenylketonuria	14	12	26
Familial hypercholesterolemia	0	18	18
Medium-chain-acyl-CoA-dehydrogenase deficiency	9	7	16
Carnitine transporter deficiency	0	6	6
Unexplained hypoglycemia	3	3	6
Biotinidase deficiency	2	3	5
Hyperphenylalaninemia	2	2	4
Isovalerianacidemia	3	1	4
Glycogenosis type I	1	2	3
Long-chain-acyl-CoA-dehydrogenase deficiency	1	2	3
Smith–Lemli–Opitz syndrome	2	1	3
Congenital hyperinsulism	0	2	2
Methylenetetrahydrofolate-reductase deficiency	0	2	2
Very-long-chain-acyl-CoA-dehydrogenase deficiency	0	2	2
Vitamins B12 deficiency	2	0	2
3-Methylcrotonyl-CoA-carboxylase deficiency	0	1	1
α-Mannosidose	1	0	1
Atypical phenylketonuria	1	0	1
Beckwith-Wiedemann syndrome	0	1	1
Galactosemia	1	0	1
Glutaraciduria type I	0	1	1
Glycogenosis type IX	1	0	1
Morbus Niemann–Pick type A	1	0	1
Non-ketotic hyperglycinemia	1	0	1
Ornithine-transcarbamylase deficiency	0	1	1
Pyruvate dehydrogenase deficiency	1	0	1
Tyrosinemia	0	1	1
X-Adrenoleukodystrophy	0	1	1
No diagnosis yet at the time of the interview.	1	4	5
Median number of medicinal products/nutritional supplements for the IEM (Q25/Q75; min/max) [N]	1 (1/2; 0/10)	1 (1/2; 0/7)	1 (1/2; 0/10)
Median number of medicinal products/nutritional supplements for other chronic conditions or as-needed medication (Q25/Q75; min/max) [N]	6 (4/8; 1/19)	5 (3/7; 1/14)	6 (3.5/8; 1/19)

* Two parents each had one child younger than 6 years old and one older than 6 years.

**Table 2:** Types and numbers of problems parents reported about their children’s medication intake. Multiple answers were possible.

Patients	Patients Aged < 6 Years (N = 40)			Patients Aged ≥ 6 Years (N = 56)		
Problems with Medication Intake	Problems [N]	Affected Patients [%]	Affected Patients [% (/13 Who Had Problems with Medication Intake)]	Problems [N]	Affected Patients [%]	Affected Patients [% (/26 Who Had Problems with Medication Intake)]
It is difficult to integrate intake into everyday life.	3	8	27	1	2	4
The intake is forgotten every now and then.	0	0	0	20	36	77
My child does not want to take the medication.	9	23	81	11	20	42
My child cannot take the medication without problems.	2	5	18	1	2	4
Total	14	33	100	33	46	100

**Table 3:** Feared and experienced adverse drug reactions (ADR) from the parents’ perspective. Multiple answers were possible.

Patients	Patients Aged < 6 Years (N = 40)		Patients Aged ≥ 6 Years (N = 56)	
ADR	Feared ADR [N] (%)	Experienced ADR [N] (%)	Feared ADR [N] (%)	Experienced ADR [N] (%)
Non-specific ADR	7 (18)	0 (0)	8 (14)	0 (0)
Gastroenterological ADR	5 (13)	7 (18)	3 (5)	7 (13)
Neurological/psychiatric ADR	3 (8)	3 (8)	5 (9)	1 (2)
Long-term side effects (with unlicensed medicinal products)	3 (8)	0 (0)	3 (5)	0 (0)
Organ damage	3 (8)	1 (3)	4 (7)	0 (0)
Cancer	1 (3)	0 (0)	1 (2)	0 (0)
Weight gain	1 (3)	0 (0)	1 (2)	2 (4)
Developmental disorders	1 (3)	0 (0)	1 (2)	0 (0)
Dental problems	1 (3)	1 (3)	0 (0)	0 (0)
Diabetes	0 (0)	0 (0)	1 (2)	0 (0)
Effects on skin, hair, nails	0 (0)	0 (0)	0 (0)	3 (5)
Fish odor	1 (3)	1 (3)	0 (0)	3 (5)
Feeling of satiety	0 (0)	1 (3)	0 (0)	0 (0)
Increased urge to urinate	0 (0)	0 (0)	0 (0)	1 (2)

**Table 4:** Expert assessment of drug-related problems of the children’s medications in relation to preparations (N = 884) and affected patients aged < 6 years (N = 45) and ≥ 6 years (N = 69).

Expert Assessment	Number of Affected Preparations [N] (%)	Number of Affected Patients Aged < 6 Years [N] (%)	Number of Affected Patients Aged ≥ 6 Years [N] (%)
**Medication appropriate**	**711 (80)**	**28 (62)**	**36 (52)**
**Medication problematic without clinical relevance**	**37 (4)**	**2 (4)**	**24 (35)**
The oral dosage form was too large for the patient’s age, according to the literature; the affected patients were able to swallow the dosage form ^a^	18 (2)	1 (2)	8 (12)
Age (patient too old) ^a^	16 (2)	2 (4)	13 (19)
Inappropriate excipient according to literature in phenylketonuria, unproblematic due to phenylalanine tolerance at occasional use ^b^	3 (0.3)	0 (0.0)	3 (4)
**Necessary medication with clinically relevant problems because no safe alternatives are available**	**26 (3)**	**8 (18)**	**7 (10)**
Age (patient too young) ^a^	4 (0.4)	1 (2)	0 (0.0)
Off-label (used for IEM *) ^b^	12 (1)	5 (11)	5 (7)
Off-label (used for other chronic indications) ^a^	8 (0.9)	3 (7)	2 (3)
**Non-necessary medication with clinically relevant problems because safe alternatives are available**	**20 (2)**	**12 (27)**	**12 (17)**
Age (patient too young) ^a^	10 (1)	8 (18)	6 (9)
Inappropriate excipient for patient’s age ^a^	4 (0.4)	4 (9)	1 (1)
Use for the wrong indication ^a^	3 (0.3)	1 (2)	3 (4)
Use despite contraindications due to comorbidities ^a^	2 (0.2)	0 (0.0)	2 (3)
Medication is not probe-compatible in patients with a probe ^b^	1 (0.1)	1 (2)	0 (0.0)
**Not assessable due to lack of specifications**	**90 (10)**	**27 (60)**	**28 (41)**
Medication for IEM *	44 (5)	16 (36)	17 (25)
Medication for other conditions	46 (5)	14 (20)	16 (23)

* IEM: inborn error of metabolism. ^a^ Medication for other conditions than inborn errors of metabolism. ^b^ Medication for inborn errors of metabolism.

## Data Availability

The data presented in this study are available on request from the corresponding author. The data are not publicly available due to ethical and privacy considerations to protect the confidentiality of participants.

## Supplementary Materials

The following supporting information can be downloaded at https://www.mdpi.com/article/10.3390/children10121873/s1, Table S1: Anatomical Therapeutic Chemical Classification (ATC-Code) of the medication used for the treatment of the inborn errors of metabolism (IEM) according to parents; Table S2: Anatomical Therapeutic Chemical Classification (ATC-Code) of the medication used for the patients’ other chronic conditions than inborn errors of metabolism; Table S3:Anatomical Therapeutic Chemical Classification (ATC-Code) of the as-needed medication Table S4: Questionnaire.
