# Supplementary material for: Parents’ Perceptions Regarding Their Children’s Medications and Expert-Assessed Drug-Related Problems in Pediatric Patients with Inborn Errors of Metabolism
Source: Children (Basel). 2023 Nov 29;10(12):1873. doi: 10.3390/children10121873 (PMC10741610; doi:10.3390/children10121873)
Supplement: Supplementary file 1 [file children-10-01873-s001.zip › children-2720717-supplementary.pdf]

Supplementary Table S1: Anatomical Therapeutic Chemical Classification (ATC-Code) of the medication used for the treatment of the inborn errors of metabolism (IEM) according to parents.

ATC-Code	Name of medication for IEM therapy	Patients aged < 6 years	Patients aged ≥ 6 years	Total
**A**	**Alimentary tract and metabolism**	**27**	**30**	**57**
A02	Drugs for acid related disorders	1	0	1
	Omeprazole	1	0	1
A03	Drugs for functional gastrointestinal disorders	0	1	1
	Simeticone	0	1	1
A05	Bile and liver therapy	1	0	1
	Ursodeoxycholic acid	1	0	1
A11	Vitamins	12	9	21
	Biotin (Vitamin B7)	3	3	6
	Cholecalciferol (Vitamin D3)	8	2	10
	Riboflavin (Vitamin B2)	0	2	2
	Thiamine (Vitamin B1)	1	0	1
	Vitamin A	0	2	2
A12	Mineral supplements	2	2	4
	Sodium selenite pentahydrate	0	1	1
	Zinc orotate	2	1	3
A16	Other alimentary tract and metabolism products	11	18	29
	L-Carnitine	5	14	19
	Nitisinone	0	1	1
	Sodium benzoate	1	1	2
	Sodium phenyl butyrate	0	1	1
	Tetrahydrobiopterin	4	1	5
	Velmanase alfa	1	0	1
**B**	**Blood and blood forming organs**	**5**	**4**	**9**
B03	Antianemic preparations	5	4	9
	Cobalamin (Vitamin B12)	1	0	1
	Ferrous glycine sulfate	3	1	4
	Folate (Vitamin B9)	1	3	4
**C**	**Cardiovascular system**	**1**	**11**	**12**
C09	Agents acting on the renin-angiotensin system	0	1	1
	Ramipril	0	1	1
C10	Lipid modifying agents	1	10	11
	Ezetimibe	0	1	1
	Pravastatin	0	7	7
	Rosuvastatin	0	1	1
	Simvastatin	1	1	2
**D**	**Dermatologicals**	**0**	**1**	**1**
D10	Anti-acne preparations	0	1	1
	Adapalene	0	1	1

**H**	**Systemic hormonal preparations, excl. Sex hormones and insulins**	**0**	**1**	**1**
H02	Corticosteroids for systemic use	0	1	1
	Hydrocortisone	0	1	1
**J**	**Antiinfectives for systemic use**	**0**	**1**	**1**
J01	Antibacterials for systemic use	0	1	1
	Phenoxymethylpenicillin	0	1	1
**M**	**Muscolo-skeletal system**	**0**	**3**	**3**
M01	Antiinflammatory and antirheumatic products	0	1	1
	Ibuprofen	0	1	1
M04	Antigout preparations	0	2	2
	Allopurinol	0	2	2
**N**	**Nervous system**	**10**	**0**	**10**
N03	Antiepileptics	2	0	2
	Levetiracetam	1	0	1
	Oxcarbazepine	1	0	1
N04	Anti-parkinson drugs	4	0	4
	Carbidopa	2	0	2
	Levodopa	2	0	2
N05	Psycholeptics	4	0	4
	Diazepam	2	0	2
	Melatonin	1	0	1
	Midazolam	1	0	1
**R**	**Respiratory system**	**3**	**0**	**3**
R05	Cough and cold preparations	1	0	1
	Dextromethorphan	1	0	1
R06	Antihistamines for systemic use	2	0	2
	Cetirizine	2	0	2
**V**	**Various**	**2**	**2**	**4**
V03	All other therapeutic products	2	0	2
	Calcium folinate	1	0	1
	Diazoxide	1	0	1
V06	General nutrients	0	2	2
	Isoleucine	0	1	1
	L-Citrulline	0	1	1
**No classification**		**29**	**39**	**68**
	Cornstarch	0	2	2
	Dietetics (Amino acid mixtures)	15	14	29
	Walnut oil	0	1	1
	Cholesterol	2	1	3
	Docosahexaenoic acid	1	0	1
	Maltodextrin	9	14	23
	Medium-chain triglycerides preparations	1	3	4
	Micronutrient preparation	1	1	2
	Modified cornstarch therapy	0	1	1
	Sugar solution	0	2	2
**No medication**		**5**	**12**	**17**

Supplementary Table S2: Anatomical Therapeutic Chemical Classification (ATC-Code) of the medication used for the patients' other chronic conditions than inborn errors of metabolism.

ATC-Code		Patients aged < 6 years	Patients aged ≥ 6 years	Total
**A**	**Alimentary tract and metabolism**	**3**	**2**	**5**
A05	Bile and liver therapy	0	1	1
A06	Drugs for constipation	1	0	1
A11	Vitamins	2	1	3
**B**	**Blood and blood forming organs**	**0**	**1**	**1**
B03	Antianemic preparations	0	1	1
**C**	**Cardiovascular system**	**0**	**3**	**3**
C08	Calcium channel blockers	0	1	1
C09	Agents acting on the renin-angiotensin system	0	2	2
**D**	**Dermatologicals**	**2**	**4**	**6**
D02	Emollients and protectives	2	0	2
D07	Corticosteroids, dermatological preparations	0	2	2
D10	Anti-acne preparations	0	2	2
**H**	**Systemic hormonal preparations, excl. sex hormones and insulins**	**0**	**1**	**1**
H03	Thyroid therapy	0	1	1
**L**	**Antineoplastic and immunomodulating agents**	**0**	**2**	**2**
L04	Immunosuppressants	0	2	2
**M**	**Musculo-skeletal system**	**0**	**2**	**2**
M01	Antiinflammatory and antirheumatic products	0	2	2
**N**	**Nervous system**	**3**	**3**	**6**
N03	Antiepileptics	2	1	3
N05	Psycholeptics	1	1	2
N06	Psychoanaleptics	0	1	1
**R**	**Respiratory system**	**1**	**8**	**9**
R01	Nasal preparation	0	2	2
R03	Drugs for obstructive airway diseases	1	3	4
R06	Antihistamines for systemic use	0	3	3
**S**	**Sensory organs**	**1**	**3**	**4**
S01	Ophthalmologicals	0	3	3
S02	Otologicals	1	0	1
**V**	**Various**	**0**	**2**	**2**
V01	Allergens	0	1	1
V06	General nutrients	0	1	1
**No classification**	**Preparations purchased in pharmacies**	**0**	**1**	**1**

Supplementary Table S3: Anatomical Therapeutic Chemical Classification (ATC-Code) of the as-needed medication.

ATC-Code		Patients aged < 6 years	Patients aged ≥ 6 years	Total
**A**	**Alimentary tract and metabolism**	**37**	**27**	**64**
A01	Stomatological preparation	4	2	6
A02	Drugs for acid related disorders	0	1	1
A03	Drugs for functional gastrointestinal disorders	5	7	12
A06	Drugs for constipation	3	0	3
A07	Antidiarrheals, intestial antiinflammatory/antiinfective agents	19	16	35
A11	Vitamins	6	1	7
**B**	**Blood and blood forming organs**	**2**	**1**	**3**
B01	Antithrombotic agents	0	1	1
B03	Antianemic preparations	2	0	2
**D**	**Dermatologicals**	**22**	**13**	**35**
D01	Antifungals for dermatological use	3	2	5
D02	Emollients and protectives	9	3	12
D03	Preparations for treatment of wounds and ulcers	3	4	7
D04	Antipruritics, incl. antihistamines, anesthetics, etc.	2	1	3
D06	Antibiotics and chemotherapeutics for dermatological use	1	0	1
D07	Corticosteroids, dermatological preparations	2	0	2
D08	Antiseptics and disinfectants	1	3	4
D10	Anti-acne preparations	1	0	1
**G**	**Genito urinary system and sex hormones**	**0**	**2**	**2**
G03	Sex hormones and modulators of the genital system	0	1	1
G04	Urologicals	0	1	1
**H**	**Systemic hormonal preparations, excl. sex hormones and insulins**	**1**	**2**	**3**
H02	Corticosteroids for systemic use	1	2	3
**M**	**Musculo-skeletal system**	**37**	**55**	**92**
M01	Antiinflammatory and antirheumatic products	37	55	92
**N**	**Nervous system**	**25**	**20**	**45**
N01	Anesthetics	1	0	1
N02	Analgesics	22	20	42
N05	Psycholeptics	2	0	2
**P**	**Antiparasitic products, insecticides and repellents**	**2**	**2**	**4**
P03	Ectoparasiticides, incl. scabicides, insecticides and repellents	2	2	4

**R**	**Respiratory system**	**98**	**182**	**280**
R01	Nasal preparation	35	44	79
R02	Throat preparations	2	22	24
R03	Drugs for obstructive airway diseases	8	1	9
R05	Cough and cold preparations	43	83	126
R06	Antihistamines for systemic use	10	32	42
**S**	**Sensory organs**	**6**	**3**	**9**
S01	Ophthalmologicals	6	3	9
**V**	**Various**	**7**	**4**	**11**
V07	All other non-therapeutic products	7	4	11
**No classification**		**63**	**44**	**107**
	**Preparations purchased in pharmacies**	**63**	**32**	**95**
	Homeopathic and anthroposophic preparations	30	22	52
	Other preparations	33	10	43
	**Preparations not purchased in pharmacies**	**0**	**12**	**12**

Supplementary Table S4: Questionnaire. The original questionnaire was conducted in German. For a better understanding of the survey, we translated the original questionnaire. ADR: Adverse drug reaction. IEM: Inborn error of metabolism.

Question	Answer
Is your child taking any medication or nutritional supplements due to the IEM?	☐ No<br>☐ Yes, medication<br>☐ Yes, nutritional supplements<br>☐ Yes, both
**Statements**<br><br>Please tell me how much you agree with each of the following statements. You can answer on a scale from 0 to 5. 0 means you totally disagree and 5 means you totally agree. Please answer spontaneously.<br><br>"My child's medication is helping her/him to get better."<br><br>"It is important to me that my child takes the medicines exactly as prescribed by the physician."<br><br>"I am afraid that my child's condition will get worse if the medication is not taken exactly as prescribed."	[0] I totally disagree<br>[1]<br>[2]<br>[3]<br>[4]<br>[5] I totally agree
**Problems with medication**	
Are there any problems with taking the medication?	☐ Yes<br>☐ No
[If yes] Where do problems arise?	☐ It is difficult to integrate intake into everyday life.<br>☐ The intake is forgotten every now and then.<br>☐ My child does not want to take the medication.<br>☐ My child cannot take the medication without problems.<br>☐ Other:
Has your child's medication ever been discontinued or paused without a doctor's order? If yes, which medication were discontinued or paused and why?	☐ Yes<br>☐ No<br><br>Medication:_[Open answer]_<br>Reason: _[Open answer]_

**ADR**	
Are you afraid of ADR from your child's medication?<br>If yes, which ones and from which medication?	☐ Yes<br>☐ No<br><br>ADR:_[Open answer]_<br>Medication: _[Open answer]_
Have any ADR been observed from your child's medication?	☐ Yes<br>☐ No<br><br>ADR:_[Open answer]_<br>Medication: _[Open answer]_
**Expert assessed drug-related problems**	
What medication is your child receiving due to the IEM?	_[Open answer]_
Does your child have any other medical conditions for which he/she is taking medication? If so, what diseases does he/she have and what medication are being taken for them?	_[Open answer]_
Do you have any other medicines at home that are intended for your child? This also means medicines that you bought at the pharmacy without a prescription.	_[Open answer]_
I will read you a few more complaints that your child may have from time to time. Do you give your child a certain medication for this? If yes, which one(s)?	☐ Cough: _[Open answer]_<br>☐ Sore throat: _[Open answer]_<br>☐ Rhinitis: _[Open answer]_<br>☐ Diarrhea: _[Open answer]_<br>☐ Vomiting: _[Open answer]_<br>☐ Abdominal pain: _[Open answer]_<br>☐ Pain/Headache: _[Open answer]_<br>☐ Fever: _[Open answer]_
Sociodemographic data	
Patients age	___ years
Patients sex	[1] male<br>[2] female<br>[3] divers
Patients diagnosis	_[Open answer]_
Parents age	__ years
Parents sex	[1] male<br>[2] female<br>[3] divers
